# Single-cycle parainfluenza virus type 5 vectors for producing recombinant proteins, including a humanized anti-V5 tag antibody

**DOI:** 10.1099/jgv.0.002061

**Published:** 2025-01-09

**Authors:** Richard E. Randall, Dan Young, Maria Pisliakova, Jelena Andrejeva, Lynsey West, Luis Rossler, Volker Morath, David Hughes, Steve Goodbourn

**Affiliations:** 1 School of Biology, Centre for Biomolecular Sciences, BMS Building, North Haugh, University of St. Andrews, St. Andrews, Fife, KY16 9ST, UK; 2 Department of Nuclear Medicine, University Clinic rechts der Isar, School of Medicine, Technical University of Munich, 81675 Munich, Germany; 3 Section for Pathogen Research, Institute for Infection and Immunity, St George's, University of London, London, SW17 0RE, UK

**Keywords:** anti-V5 antibody, expression vectors, parainfluenza virus type 5, persistence

## Abstract

Parainfluenza virus type 5 (PIV5) can cause either persistent or acute/lytic infections in a wide range of mammalian tissue culture cells. Here, we have generated PIV5 fusion (F)-expressing helper cell lines that support the replication of F-deleted viruses. As proof of the principle that F-deleted single-cycle infectious viruses can be used as safe and efficient expression vectors, we have cloned and expressed a humanized (Hu) version of the mouse anti-V5 tag antibody (clone SV5-Pk1). We show that multiple different cell lines can be infected and express high levels of the Hu anti-V5 antibody, with Chinese hamster ovary cells expressing 20–50 mg l^−1^ after 5 days when cells were grown to a density of ~1×10^6^ cells per millilitre at the time of infection. We suggest that PIV5-based vectors may be further developed to produce recombinant proteins both *in vitro* and *in vivo*.

## Introduction

There is a plethora of different vector systems used to express recombinant proteins in eukaryotes both *in vitro* and *in vivo*, including a wide variety of virus-based systems [1]. We are beginning to develop and evaluate the potential use of parainfluenza virus type 5 (PIV5)-based vectors in the expectation that in certain situations, they may have significant advantages over other systems. This is based on a variety of properties of PIV5 (discussed below), including its ability to immediately establish persistent infections in most mammalian cells.

PIV5 [previously known as simian virus 5 (SV5); species *Mammalian rubulavirus 5*] is a member of the *Orthorubulavirus* genus in the family *Paramyxoviridae*, order *Mononegavirales*. PIV5 has been isolated from a wide variety of mammals, including humans, monkeys, dogs, cattle and pigs [2,4], and a very closely related virus, Alston virus, has been isolated from pteropid bats [5].

Like other paramyxoviruses, PIV5 has a lipoprotein envelope through which protrude the haemagglutinin-neuraminidase (HN) and fusion (F) proteins, which are involved in virus entry and cell–cell F, as well as in some strains a small hydrophobic (SH) protein. The matrix (M) protein is located on the inner surface of the envelope, where it structurally links the envelope glycoproteins to the viral ribonucleocapsid (vRNP). The vRNP consists of the non-segmented, single-stranded negative-sense RNA genome encapsidated by the nucleoprotein (NP). Associated with the nucleocapsid is the viral RNA-dependent RNA polymerase (vRdRp), which comprises the large (L) protein and the phosphoprotein (P). The V protein, which is the viral IFN antagonist, is also found in the virion and is associated with the vRNP. The genome has 15 246 bases, with 7 genes (3′-NP-V/P-M-F-SH-HN-L-5′) that encode the 8 viral proteins (the V and P proteins are made from differentially transcribed mRNAs from the same V/P gene). The non-coding leader (Le) and trailer (Tr) sequences are located at their 3′ and 5′ ends, respectively (reviewed in ref [6]). The vRdRp recognizes the Le promoter to drive the expression of viral mRNAs through recognition of *cis*-acting gene start (Gs) and gene end (Ge) elements that encompass each gene. As the vRdRp transcribes the genome, there is a progressive chance that it will disengage from the template, resulting in a transcriptional mRNA gradient with the NP mRNA most abundant, due to its proximity to the 3′ promoter, and the L mRNA the least abundant. The vRdRp also initiates the replication of full-length 5′−3′ antigenomes from Le that serve as templates for the synthesis of full-length 3′−5′ genomes from Tr on the antigenome (for a general review of the molecular biology of paramyxoviruses, including PIV5, see [6,7]).

Different isolates of PIV5 can have either acute/lytic or persistent phenotypes; those with a persistent phenotype can immediately establish persistent infections in tissue culture cells without them going into crisis or causing cytopathic effects and with the cells being able to be readily passaged [8,9]. In persistently infected cells, virus transcription fluxes between repressed and active states, leading to the continuous long-term production of infectious virus. Critical aas in the P protein determine whether an isolate has a persistent or acute/lytic phenotype. Single nt mutations resulting in aa substitutions at these critical aas can change the phenotype of a particular isolate from a persistent to acute/lytic phenotype and vice versa. For example, the W3 strain of PIV5 has a serine at position 157 (S157) in P and has a persistent phenotype, but a single nt change resulting in substitution by phenylalanine (F157) switches the virus to an acute/lytic phenotype. Because S157 is phosphorylated during infection, it has been suggested that phosphorylation by a cellular kinase(s) serves as the switch between lytic and persistent phenotypes. We have suggested that this ability to readily switch from acute/lytic to persistent phenotypes has been selected during evolution as, whilst acute/lytic variants would replicate and spread more rapidly, persistent variants may result in some infected individuals becoming persistently infected, thereby acting as reservoirs for the virus within a susceptible population [8,9].

An attenuated strain of PIV5 has also been successfully used, without safety issues, for over 40 years as a component of vaccines against kennel cough [10]. Given the widespread use of this vaccine, a number of fully replication-competent recombinant PIV5 vectors are currently in development as human intranasal vaccines [11] including vaccines directed against SARS-CoV-2 [12] and respiratory syncytial virus (RSV)[13]. Following their ability to generate robust adaptive immune responses in experimental animals, and the successful completion of phase 1 human trials (NCT05281263 and NCT04954287), both the PIV5-based SARS-CoV-2 and the PIV5-based RSV vaccines – at the time of writing – have now entered phase 2 clinical trials (NCT05736835 and NCT05655182, respectively).

Given the ability of PIV5 to infect cells from multiple species and to establish acute/lytic and persistent infections in these cells, we have begun to develop a number of PIV5-based vectors as an expression system for producing recombinant proteins *in vitro* and potentially *in vivo*. In this study, we have cloned a humanized version of the mouse anti-V5 epitope (which was originally called the Pk epitope; clone SV5-Pk1 [14,17]) tag antibody, which is widely used in molecular biology, into a single-cycle PIV5-based expression vector, in which the F gene has been deleted (it will thus only produce infectious virus in F-expressing helper cell lines) and show that it can be used to produce high levels of the antibody in a variety of mammalian cells.

## Methods

### Cells and viruses

Monolayers of BSRT7 [18], Vero (ECACC 84113001) and A549 (ECACC 86012804) cells were grown in 25 cm^2^ or 75 cm^2^ cell culture flasks, in Dulbecco’s modified Eagle medium (DMEM) supplemented with 5% (BSRT7) or 10% (v/v) FBS at 37 °C. Suspension Chinese hamster ovary (CHO) cells (ECACC 85050302) were grown in CD CHO medium (Gibco 10743-029). To create cells with inducible F expression, the sequence of the PIV5 (W3A) F ORF was codon optimized (GenSmart Codon Optimization tool, GenScript) and synthesized and cloned (GenScript) using the lentiviral transfer vector pLVX-TetOne-Puro (Creative Biogene, OVT2364), which includes separate promoters for constitutive expression of Pac (for puromycin resistance) and a doxycycline (Dox)-regulated promoter for inducible expression of F. Lentiviral vectors were generated and used to transduce target cells. Puromycin-resistant cells (10 µg ml^−1^ for Vero cells and 2 µg ml^−1^ for BSR-T7, A549 and CHO cells) were tested for inducible expression of PIV5 F. Stocks of the PIV5 reporter viruses were propagated and titrated in Vero, and for F-deleted viruses, in Vero.F cells, in DMEM supplemented with 2% (v/v) FBS in the presence or absence of Dox (0.5 µg ml^−1^), as appropriate.

A gene encoding a secreted form of the bovine ephemeral fever virus glycoprotein (BEFV Gt), which had its predicted transmembrane and cytoplasmic tail replaced by the V5 epitope tag [19], was also subcloned into pLVX-TetOne-Puro vector. Lentiviral vectors and an A549 cell line that inducibly expresses a secreted form of BEFV Gt were produced in the same manner.

### Construction and rescue of mCherry and GFP reporter viruses with acute/lytic or persistent phenotypes

PIV5.S157.mCherry has been previously described [9]. PIV5.F157.mCherry was derived from PIV5.S157.mCherry by replacement of the AgeI–SmaI fragment of PIV5.S157.mCherry with the equivalent fragment from a viral genome encoding F157. The F gene was removed (deleting between PIV5 nt 4501 and 6224) from both PIV5.S157.mCherry and PIV5.F157.mCherry by PCR-directed mutagenesis and Gibson assembly to generate PIV5ΔF.S157.mCherry and PIV5ΔF.F157.mCherry. A derivative of PIV5ΔF.F157.mCherry with an N100D change in the P gene (PIV5ΔF.F157.D100.mCherry) was generated by replacement of the AgeI–SmaI fragment of PIV5ΔF.F157.mCherry with the equivalent fragment from a viral genome containing F157 and D100. A synthetic gene encoding the heavy and light chains of the anti-V5 antibody separated by a coding sequence for *Thosea asigna virus* (TaV) 2A was cloned into PIV5ΔF.F157.D100.mCherry. The sequences of fully assembled genomic plasmids were confirmed using Nanopore sequencing (Source Bioscience)

Viruses were rescued as originally described by He *et al.* for PIV5 (SV5) [20] by co-transfecting 1 µg of ‘virus rescue’ plasmids with pCAGGS-based helper plasmids directing the synthesis of PIV5-NP (100 ng), PIV5-P (100 ng) and PIV5-L (500 ng) into 6-well dishes containing ~1×10^6^ BSRT7 cells using Lipofectamine LTX/PLUS (Invitrogen, 15338100). Recovery was monitored by observing the expression of mCherry or GFP, and stocks of the virus were amplified by two successive passages at low m.o.i. in Vero cells or Vero.F cells as described above.

### Surface plasmon resonance spectroscopy

The affinity of interaction between different anti-V5 antibody variants and the V5 tag was assessed using the surface plasmon resonance (SPR) spectroscopy measured on a Biacore T200 SPR biosensor system (GE Healthcare, Chicago, IL). A purified protein with a C-terminal V5 tag was conjugated onto a C1 sensor chip (GE Healthcare) using amine coupling chemistry according to the manufacturer’s instructions. As a control, the same purified protein without the V5 tag was immobilized onto the reference flow channel to control for nonspecific binding. Experiments were conducted at 25 °C in running buffer (HBS-P) containing 10 mM HEPES buffer (pH 7.4), 150 mM NaCl, 0.05% (v/v) surfactant P20 and 1 mM MgCl_2_. To measure the individual *K*_D_ of the anti-V5 antibody:V5 tag complex, each anti-V5 antibody was diluted in a tenfold dilution series from 3.5 nM to 0.35 pM in HBS-P and injected (in cycles of increasing concentration) onto the chip surface at a flow rate of 5 µl min^−1^, until the V5 tag was saturated. Subsequently, a dissociation period of 5000 s was used, after which the sensor chip surface was regenerated between each injection through sequential 360-s injections of 200 nM glycine/HCl pH 2.8 and 50 mM HCl. Data were analysed using the Biacore Evaluation software, and the association and dissociation kinetics of binding were measured.

### Visualization of mCherry-expressing cells and plaques and immunofluorescence

Infected cells expressing mCherry and mCherry-positive viral plaques in unfixed but washed (1× with complete PBS) cells were visualized using an EVOS M5000 Cell Imaging System with Plan Achromat 4, 10 or 20× objectives. Procedures for immunofluorescence on fixed and permeabilized cells grown on coverslips have been described previously [21,22]. The primary antibodies used were the mouse mAbs F-1a and NP-1a [17] or the humanized anti-V5 antibody described here; the secondary antibodies were either Cy5-conjugated goat anti-mouse IgG (abcam, ab6563) or Texas Red-conjugated horse anti-mouse IgG (Vector Laboratories, T1-2000) and FITC-conjugated goat anti-human IgG (abcam, AB6785) for detection. Where appropriate, the cell nuclei were visualized by staining with DAPI.

### Solid matrix-antibody-antigen complexes

Preparation of a 10% (w/v) suspensions of fixed and killed Cowan strain A of *Staphylococcus aureus* (St.A.), the capture of antibodies onto St.A. and the generation of solid matrix-antibody-antigen (SMAA) complexes have previously been described [23]. Briefly, A549 cells with Dox-inducible expression of soluble V5-tagged BEFV Gt, grown in 75 cm^2^ flasks with 5 ml of DMEM +2% FCS, were treated with Dox, and the culture media were harvested 96 h post-induction. The medium was clarified by centrifugation, and 1.5 ml of media was incubated with 20 µl (w/v) of St.A. alone, or 20 µl of St.A. saturated with the original mouse anti-V5 antibody, the mouse/human chimeric antibody (Hu1) or the nine humanized anti-V5 antibodies [2,10] as previously described [23]. The resulting SMAA complexes were washed three times by centrifugation and resuspension in PBS-A. After the final wash, proteins bound to the St.A. or captured in SMAA complexes were eluted into 50 µl of disruption buffer (6 M urea, 5% (w/v) SDS and 2 M 2-mercaptoethanol) and heated at 100 °C for 5 min. Twenty microlitres of each sample were electrophoresed through an SDS-PAGE that was subsequently stained with Coomassie brilliant blue. The equivalent of 1 µl per sample was also immunoblotted on nitrocellulose filters using a mouse anti-V5 antibody directly conjugated to HRP (Bio-Rad, MCA1360P) for detection by chemiluminescence.

### Antibody production and purification

The culture medium from 200 ml of CHO.F cells infected with PIV5ΔF.F157.D100.hu_V5.mCherry was harvested, the cells were pelleted by centrifugation and the supernatant was incubated overnight with 1 ml of protein G Sepharose (GE Healthcare, Protein G Sepharose 4 fast flow, 17-0618-01) at 4 °C, with rotation. The beads were pelleted, resuspended in 5 ml of the supernatant, packed into a 5 ml disposable column (G-Biosciences, 786-169) and washed with five column volumes of PBS. Bound antibody was eluted with 0.1 M glycine/HCL pH 2.8, and fractions containing the antibody were pooled and immediately neutralized with 1 M Tris/HCL pH 8.6.

## Results

### Humanization of the anti-V5 antibody

**Fig. 1.**
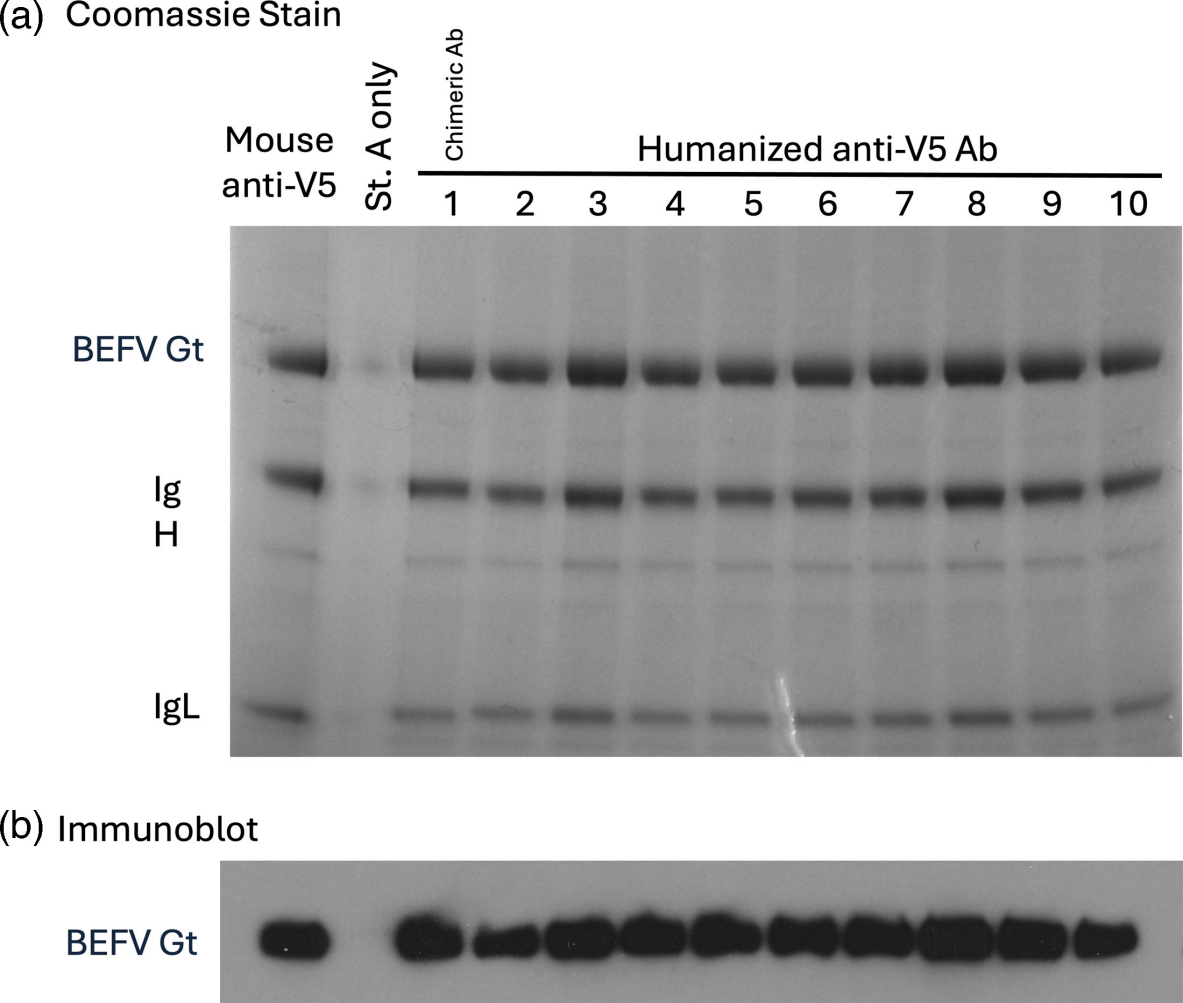
Humanized anti-V5 monoclonal antibodies recognize the V5 tag epitope. All the humanized anti-V5 antibodies recognize the V5 tag and can capture a V5-tagged soluble form of BEFV Gt onto SMAA complexes. To generate SMAA complexes, the V5-tagged BEFV Gt in the culture medium of Dox-induced A549_BEFV_Gt cells was captured onto St.A. alone (control) or St.A. saturated with the original mouse anti-V5 antibody, the mouse/human chimeric anti-V5 antibody (Hu1) or the nine humanized anti-V5 antibodies (Hu2-10). Proteins bound to the SMAA complexes were resolved using SDS-PAGE and visualized by Coomassie brilliant blue staining of the resulting PAG (panel a). V5-tagged BEFV Gt in the SMAA complexes was identified by immunoblot analysis using an HRP-conjugated mouse anti-V5 antibody (panel b).

The mouse monoclonal epitope-specific anti-V5 antibody is used in numerous immunologically based techniques and is widely available commercially. However, in certain situations, it can be advantageous to use an anti-V5 antibody from another species, and indeed, such anti-V5 antibodies can be purchased. Nevertheless, we took the view that in these situations, it might be better to use different species of anti-V5 monoclonal antibodies that have the same, or similar, binding properties to the high-affinity original mouse anti-V5 mAb (clone SV5-Pk1). Using a commercial antibody humanization service (LakePharma, Belmont, CA 94002, USA), we therefore had the original mouse anti-V5 antibody (clone SV5-Pk1 [15]) humanized. A chimeric antibody in which the mouse variable heavy and light chain domains were cloned onto human constant chain domains, and nine humanized variants, based on combinations of three humanized heavy chains and three humanized light chains, of the anti-V5 antibody were generated and purified antibody supplied, together with details of their aa sequence. Initially, to determine whether the humanized anti-V5 antibodies still recognized the V5 epitope tag, we used them to capture (pull down) a V5-tagged soluble form of the BEFV Gt [19] into SMAA complexes [23]. As can be seen from Fig. 1, all the humanized anti-V5 antibodies recognized and captured the V5-tagged BEFV Gt.

**Fig. 2.**
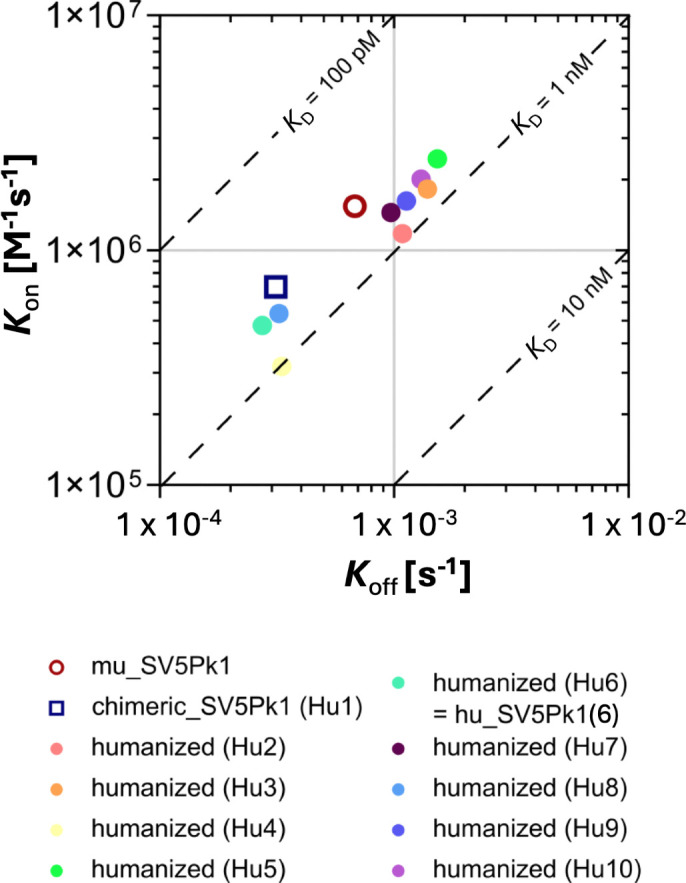
Humanized anti-V5 mAbs retain very high binding affinity for the V5 epitope tag. The relative binding affinity of the humanized anti-V5 antibodies was compared to the original mouse anti-V5 antibody as measured by SPR.

We next measured the relative affinities of the humanized antibodies for the V5 tag using SPR spectroscopy (Fig. 2). All humanized antibodies retained very high affinity binding for the V5 tag (*K*_D_=0.57–1.04 nM). The highest affinity antibody (Hu6=hu_SV5-Pk1 [6]; *K*_D_=572 pM; *k*_off_=2.74×10^−4^ s^−1^ M^−1^; *k*_on_=4.79×10^5^ s^−1^), which was characterized by the slowest *k*_off_ rate, showed binding kinetics similar to the original mouse anti-V5 antibody (SV5-Pk1; *K*_D_=441 pM; *k*_off_=6.79×10^−4^ s^−1^; *k*_on_=1.54×10^6^ s^−1^ M^−1^).

### Rescue of PIV5 F-deleted viruses

**Fig. 3.**
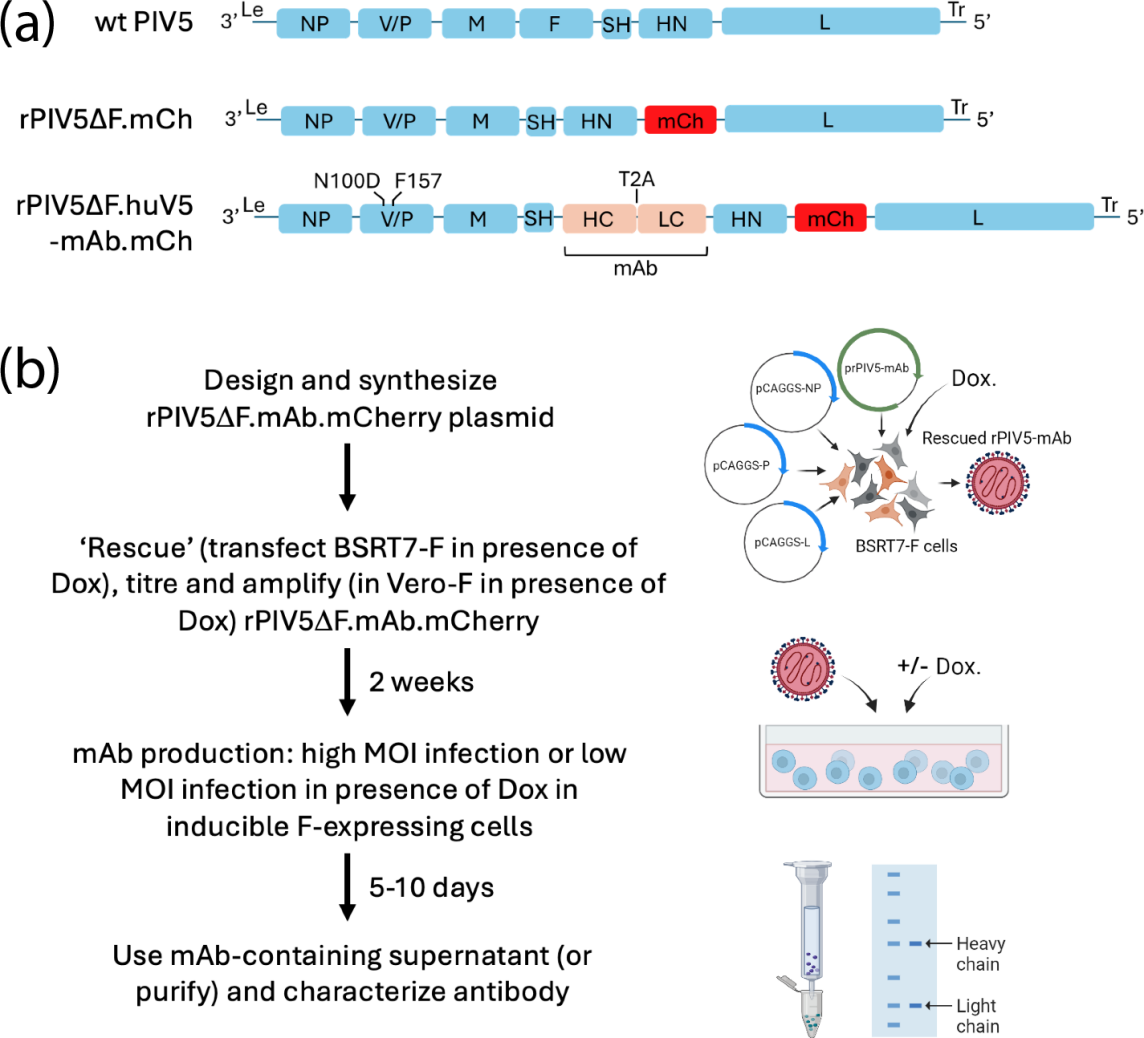
PIV5-based vectors and workflow for the production of mAb. (**a**) Schematic representation of rPIV5 vectors showing wt PIV5 (top), recombinant PIV5 in which the entire F protein gene has been deleted and an ORF that expresses mCherry (mCh) has been introduced downstream of HN (rPIV5ΔF.mCh; middle). Vectors that express P with Ser at position 157 (S157 – persistent phenotype) and Phe at position 157 (F157 – acute/lytic phenotype) were created. rPIV5ΔF.huV5-mAb.mCh (bottom) in which the heavy chain (HC) and light chain (LC) genes encoding the humanized V5 mAb have been inserted where the deleted F gene would have been located. HC and LC genes are separated by a T2A sequence, meaning they are expressed as a single mRNA but translated as two separate proteins. Additional modifications include P-N100D (to mutate the natural V5 epitope), and residue 157 in P is Phe (F157) to ensure consistent expression of viral genes (including huV5 mAb genes). (**b**) mAb expression workflow. Antibody genes are ligated into a plasmid containing rPIV5ΔF.mAb.mCh. ‘Rescue’ and ‘amplification’ of rPIV5-based vectors require BSRT7-F and Vero-F cell lines, respectively. Stable BSRT7-F and Vero-F cell lines were created by transduction with lentivirus carrying Dox-inducible PIV5-F (to *trans*-complement F; see also Fig. 4). Infectious virus is ‘rescued’ by transfection of BSRT7-F cells with prPIV5ΔF.mAb.mCh along with pCAGGS helper plasmids that express NP, P and L. Dox is added and mCherry expression is monitored (typically 5–7 days). Rescued virus is quantified on Vero-F cells (by plaque assay or flow cytometry), amplified and re-quantified. Antibody-producing cells of choice (e.g. CHO, HEK293 and Vero) can be infected at high m.o.i. (e.g. 10 p.f.u. per cell) to ensure all cells are producing mAb (monitored by mCherry expression). Alternatively, inducible PIV5-F-expressing antibody-producing cells can be used, meaning that low m.o.i. values are permitted; here, Dox is added at the time of infection, and ~24 h later, all cells are positive. The supernatant is usually collected between 5 and 10 days post-infection and used directly in immune-based experiments, or the mAb can be purified. Parts of this figure were created in BioRender.

**Fig. 4.**
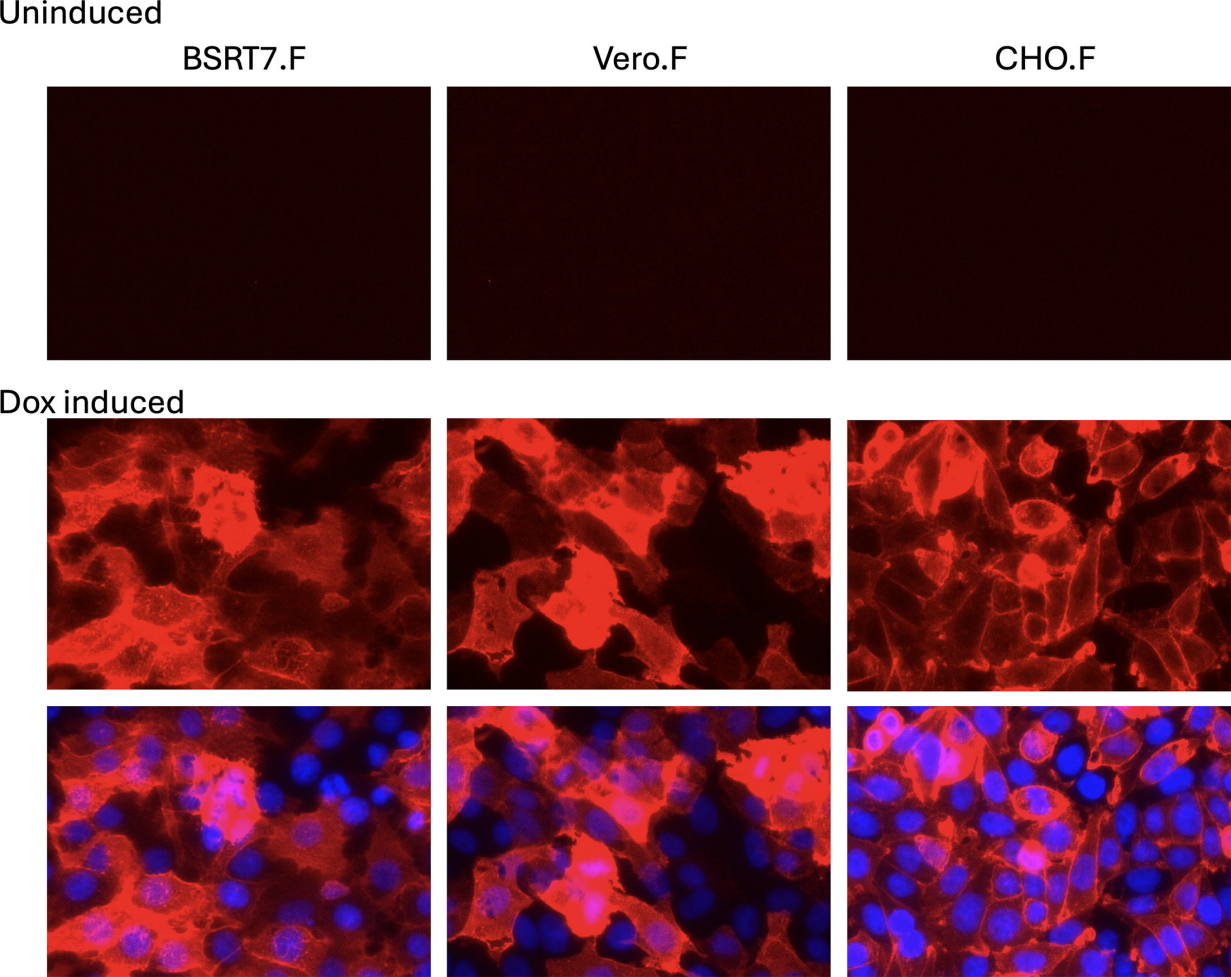
Dox-inducible expression of the PIV5 F protein. BSRT7, Vero and CHO cell lines with Dox-inducible PIV5-F expression were generated by lentiviral transduction with pLVX.F lentivirus and selection with puromycin. Confluent monolayers of the resulting BSRT7.F, Vero.F and CHO.F were, or were not, treated with Dox (1 µg ml^−1^) for 36 h and indirectly immunostained with an anti-F mAb [17] and detected using anti-mouse IgG antibody conjugated with Texas Red fluorophore. Cell nuclei in Dox-induced cells were visualized by DAPI staining.

We are currently developing a variety of PIV5-based vectors, based on the W3 strain of PIV5 (GenBank accession number JQ743318) as platforms for the expression of recombinant proteins both *in vitro* and *in vivo*. From a safety point of view, we have created single-cycle vectors (non-transmissible) in which the F gene is deleted (see Fig. 3 and below). To rescue such viruses, the F protein must be provided in trans. To do this, we used the pLVX lentivirus vector, in which the expression of the F gene was under the control of a Dox-inducible promoter. We have noticed that the expression of intronless cDNAs from cytoplasmic-replicating viruses such as PIV5 is often poor when using nuclear-expressing lentiviral vectors. We have found that codon-optimizing cDNAs improve the expression as this not only optimizes codon usage but it also removes cryptic splice sites. Codon-optimized F was cloned using pLVX, and F-expressing lentiviruses were used to transduce BSRT7, Vero and CHO cells. After puromycin selection, no expression of F was observed in cells in the absence of Dox induction. However, in the presence of Dox, high-level expression of F in most cells was achieved without the need for cell cloning (Fig. 4). However, in the case of CHO.F suspension cells, any cell that did not express the F protein was removed by panning the cells using an anti-F mAb as previously described [24].

**Fig. 5.**
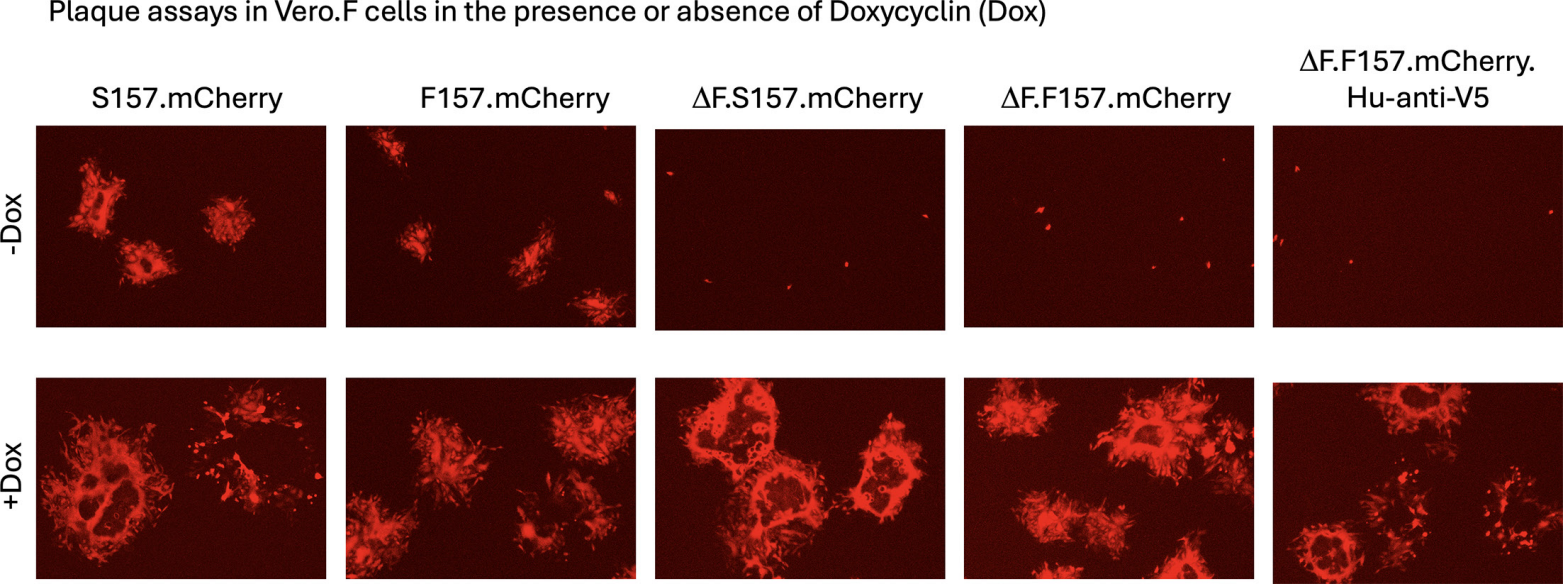
PIV5ΔF is non-transmissible. Confluent Vero-F cells in 6-well plates were infected with ~50 p.f.u. per well with the indicated virus in the presence or absence of Dox (1 µg ml^−1^) and overlayed with a medium containing carboxymethyl cellulose. Images were taken at 4 days post-infection.

Next, we deleted the F gene from both PIV5.S157.mCherry and PIV5.F157.mCherry and rescued the F-deleted viruses in BSRT7.F cells (Fig. 3). High-titre virus stocks of PIV5ΔF.S157.mCherry and PIV5ΔF.F157.mCherry (>1×10^8^ p.f.u. ml^−1^) were subsequently made in Vero.F cells. Whilst both PIV5ΔF.S157.mCherry and PIV5ΔF.F157.mCherry readily formed plaques on Vero.F cells in the presence of Dox, no plaques were observed in the absence of Dox, although, as would be expected, there were individual mCherry-positive cells, but there was no virus spread within the monolayers (Fig. 5). Importantly, the plaque sizes of PIV5ΔF.S157.mCherry, PIV5ΔF.F157.mCherry and their non-F-deleted parental viruses were similar in Dox-induced Vero.F cells (Fig. 5). However, the non-F-deleted parental viruses formed larger plaques on Vero.F cells in the presence of Dox; presumably, cell-to-cell spread occurred more rapidly if cells are already positive for F upon infection. Together, these data demonstrate that the deletion of F from the genome of PIV5 resulted in a single-cycle virus unable to spread cell-to-cell but that F can be supplied in trans to enable virus propagation.

### Expression of humanized anti-V5 antibody in PIV5 F-deleted vectors

**Fig. 6.**
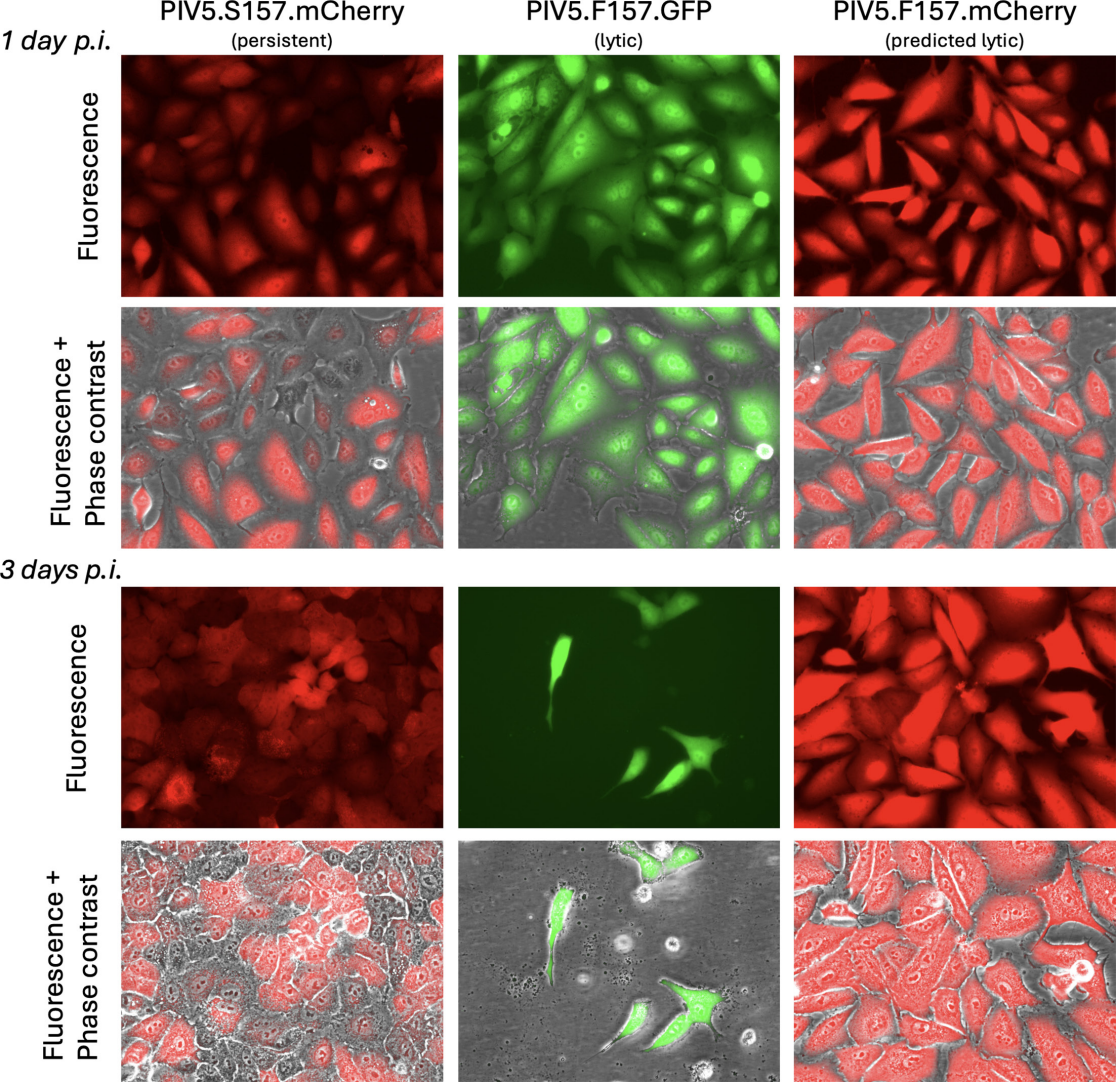
Characterization of rPIV5 reporter viruses expressing either mCherry or GFP. Recombinant viruses were constructed with either a serine (S157, associated with a persistent phenotype) or a phenylalanine (F157, associated with an acute/lytic phenotype) at position 157 in the PIV5 P [8,9] and either a GFP or mCherry reporter gene. Monolayers of A549 cells were, or were not, infected with 5 p.f.u./cell with the indicated virus. mCherry and GFP fluorescence images and phase-contrast images of infected monolayers were taken at 1 and 3 days p.i.

To investigate the potential use of PIV5ΔF as expression vectors, in this study, we cloned a codon-optimized synthetic humanized anti-V5 tag gene corresponding to hu_SV5-Pk1 [6] into the PIV5ΔF.F157.mCherry vector (Fig. 3). The reason for using PIV5ΔF.F157.mCherry vector was that although it was predicted to have an acute/lytic phenotype (because it has a phenylalanine at position 157 in the P protein) for reasons that remain unclear, the majority of cells infected with PIV5.F157.mCherry did not die by 3 days post-infection (p.i.), in contrast to cells infected with PIV5.F157.GFP, which expresses GFP rather than mCherry (Fig. 6). Furthermore, at 3 days p.i., the level of mCherry expression was higher in cells infected with PIV5.F157.mCherry than in cells infected with PIV5.S157.mCherry, which has a persistent phenotype; presumably, this is because virus transcription and replication are not switched off in cells infected with PIV5.W3A with phenylalanine at position 157 in contrast to variants with a serine at position 157 [8,9]. We considered that this unexpected property may prove useful for recombinant protein expression as the PIV5.F157.mCherry vector would lead to high levels of expression in cells that could tolerate infection for a longer duration, leading to high yields.

To facilitate the production of separate heavy and light chains (necessary for the assembly of functional antibody), we cloned the T2A cleavage site from TaV [25,26] between the heavy and light chain sequences. Also, because the mouse anti-V5 (SV5-Pk1) monoclonal antibody binds the P and V proteins of PIV5 [17] (the V5 epitope tag is derived from these proteins [14]), we modified PIV5ΔF.F157.mCherry to prevent this interaction. We had previously shown that a substitution of an asparagine (N) to aspartic acid (D) at position 100 in P and V abolished the interaction of the anti-V5 antibody with P and V ([27]; see also below). Therefore, we modified the PIV5ΔF.F157.mCherry to have an aspartic acid at position 100, creating PIV5ΔF.F157.D100.mCherry. We subsequently cloned the humanized anti-V5 antibody (hu_SV5-Pk1 [6]) gene into this vector, creating PIV5ΔF.F157.D100.hu_V5.mCherry (Fig. 3). Importantly, high-titre stocks of PIV5ΔF.F157.D100. hu_V5.mCherry could be generated in Vero.F cells (~1×10^8^ p.f.u. ml^−1^).

**Fig. 7.**
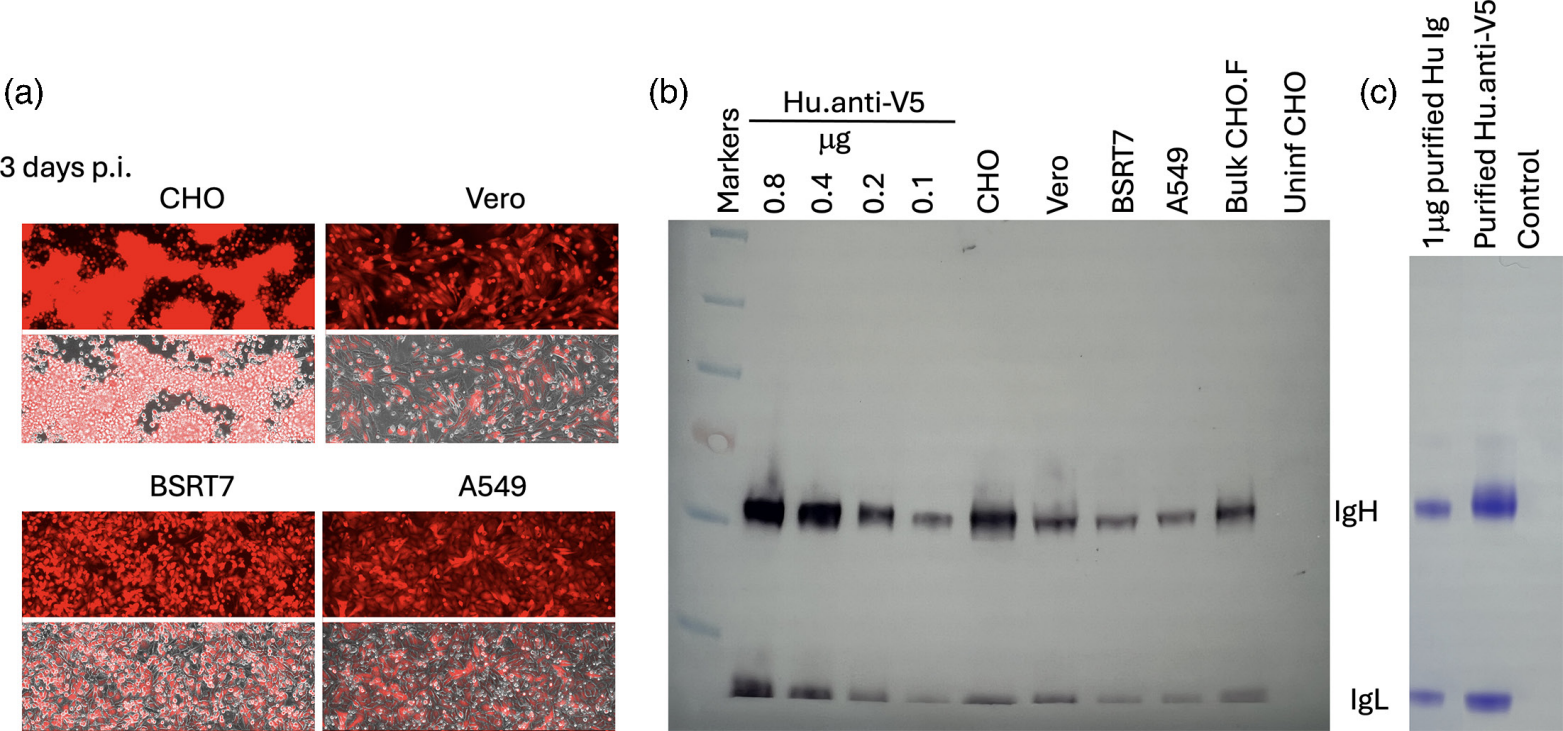
Recombinant PIV5ΔF vectors can be used to express mAbs. (a) Confluent monolayers of CHO, Vero, BSRT7 and A549 cells grown in 25-cm flasks were infected at a high m.o.i. (5 to 10 p.f.u./cell) with PIV5ΔF.F157.D100. hu_V5.mCherry. After 2-h absorption, the medium was replaced with either chemically defined CHO medium (CHO cells) or DMEM with 2% (v/v) ultra-low Ig calf serum (Vero, BSRT7 and A549 monolayers). mCherry and phase-contrast images of live cells were captured using an EVOS M5000 microscope. Images at 3 days p.i. are shown; the top part of the image shows mCherry fluorescence only, whilst the bottom shows mCherry fluorescence combined with the phase-contrast image. (b) Estimation of the amount of antibody released into the medium of CHO, Vero, BSRT7 and A549 cells, described in (a) and from a 200-ml suspension CHO.F cells (bulk CHO.F), grown to a density of 1×10^6^ cells per millilitre, which were infected with 0.1 p.f.u./cell and cultured in the presence of Dox for a further 5 days. Culture media (5 µl) from each were immunoblotted, and antibody was detected using goat anti-human antibodies conjugated to alkaline phosphatase (abcam, ab6859). At the same time, 0.8, 0.4, 0.2 and 0.1 µg of purified Hu.anti-V5 antibody were analysed. Medium from uninfected CHO cells (Unif CHO) served as the negative control. (c) Coomassie brilliant blue staining of 1 µl of purified Hu.anti-V5 antibody from the 200 ml CHO.F culture described in (b) alongside 1 µg of purified and quantified Hu.anti-V5 antibody. Control is CHO medium from uninfected CHO.F culture that was purified on a protein G beads in parallel.

To investigate whether the Hu.anti-V5 antibody was produced by cells infected with PIV5ΔF.F157.D100.hu_V5.mCherry, confluent monolayers of BSRT7, Vero, A549 and CHO cells were infected at a high m.o.i. with PIV5ΔF.F157.D100.hu_V5.mCherry, and infection was monitored by mCherry expression (Fig. 7a). All cells were maintained in growth media and at 5 days p.i., the medium was harvested and the amount of Hu.anti-V5 antibody secreted was estimated by immunoblot analysis (Fig. 7b). All cell lines were capable of producing Hu.anti-V5, and as expected, CHO cells, which are widely used in industry to produce recombinant proteins due to their propensity for high levels of heterologous protein expression [28], produced the highest amount of the Hu.anti-V5 antibody, equivalent to ~30–50 mg l^−1^. Nevertheless, Hu.anti-V5 was also secreted into the medium of other infected cells, producing approximately the equivalent of 10–20 mg l^−1^, as calculated from the amount of antibody secreted into 5 ml of medium harvested from infected confluent monolayers of Vero, A549 and BSRT7 cells grown in 75 cm^2^ flasks at 5 days p.i.

To produce large amounts of recombinant proteins from CHO cells, they are usually grown in suspension to high densities (as high as 1×10^7^ to 2×10^8^ cells per millilitre, depending on the method of culture and recombinant protein being produced [29,30]). To infect such densities at high m.o.i. (to ensure all cells are infected), very large amounts of virus would be required (for example, 1 l of cells at 1×10^7^ cells per millilitre to be infected at m.o.i. of 10 would require 1×10^11^ p.f.u.). Whilst entirely feasible for rPIV5 vectors, we considered taking advantage of the fact that PIV5ΔF will readily spread if F is provided in trans, meaning significantly less virus would be required to produce high levels of recombinant antibody (Fig. 3). We therefore inoculated a 200-ml culture of CHO.F cells, grown to a density of 1×10^6^ cells per millilitre, with 0.1 p.f.u./cell of PIV5ΔF.F157.D100.Hu_V5.mCherry in the presence of Dox. By 2 days p.i., all cells were positive for mCherry, indicating that the virus had spread throughout the culture. They were cultured for a further 3 days, after which the supernatant was collected. The amount of antibody in the culture medium was again estimated by immunoblot analysis (Fig. 7b, bulk CHO.F) and shown to be equivalent to ~20–40 mg l^−1^. Antibody in the culture medium was then purified on protein G Sepharose (Fig. 7c). Without significant optimization of the procedure, ~2 mg of purified antibody was obtained from 200 ml of culture medium. In addition to the production of the Hu-anti-V5 antibody, infectious single-cycle PIV5ΔF.F157.D100.hu_V5.mCherry was also produced, with titres of ~2×10^7^ p.f.u. ml^−1^.

**Fig. 8.**
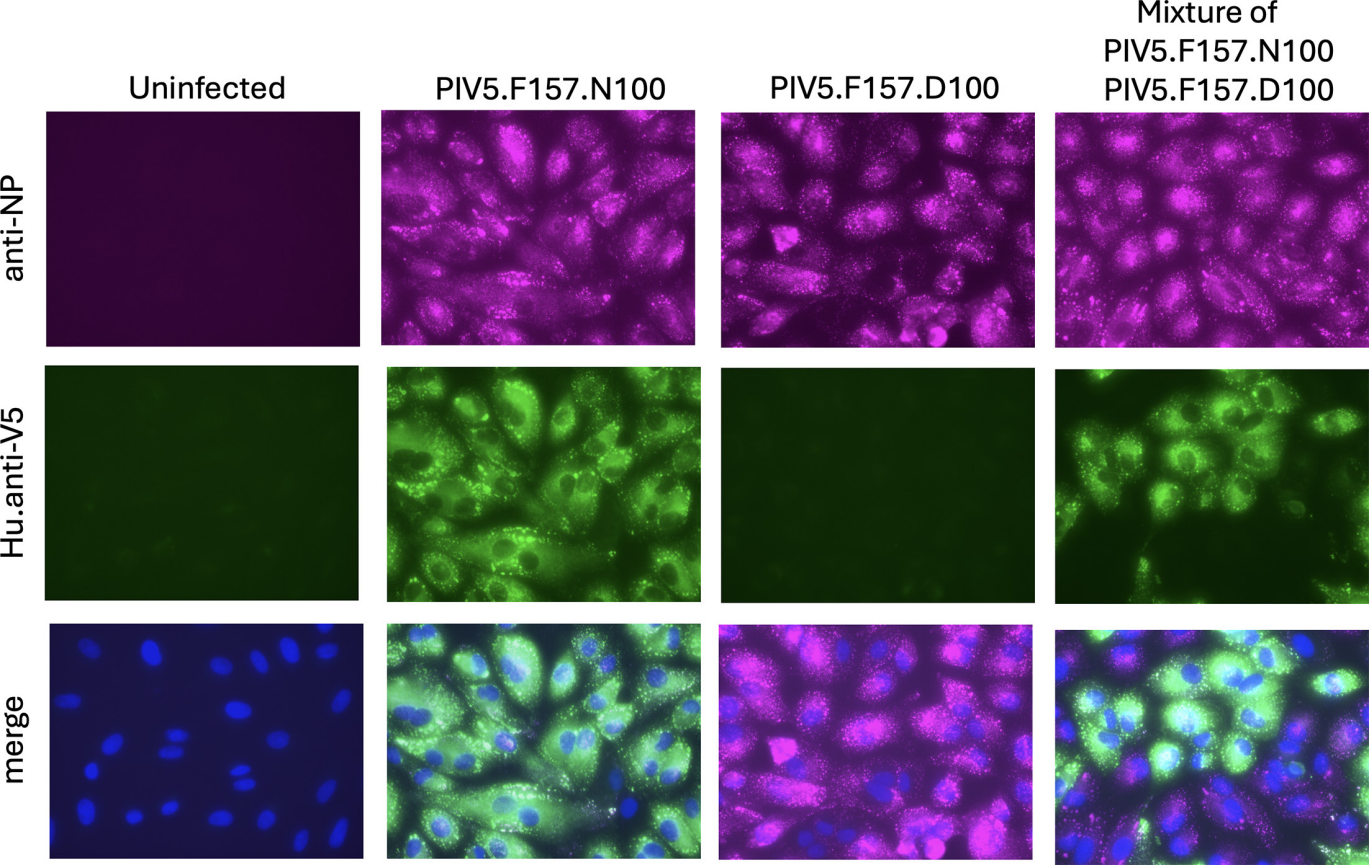
Human anti-V5 mAbs specifically recognize the V5 tag. The V5 tag is derived from an epitope within the PIV5 P and V proteins [17]. Mutation of asparagine 100 to aspartic acid abolished the recognition by anti-V5 mAbs [27]. Hu.anti-V5 antibody (hu_SV5-Pk1) recognizes the parental PIV5 strain W3A that has asparagine (N) at position 100 in the P and V proteins, but not a variant with an aspartic acid at position 100. Monolayers of Vero cells grown in 25 cm^2^ flasks were, or were not, infected at a high m.o.i. with PIV5.W3A (N100) or PIV5.W3A (D100). After 2-h absorption, the cells were trypsinized and were, or were not, mixed together prior to seeding onto coverslips in 24-well dishes. At 24 h p.i., the cells were fixed and indirectly immunostained with a mouse anti-NP mAb and the hu_anti-V5 antibody. The anti-NP mAb and the hu_anti-V5 antibody (1:1000 dilution of purified Hu.anti-V5 antibody at 0.3 mg ml^−1^) were mixed prior to immunostaining, as were the Cy5-anti-mouse antibody and the FITC-anti-human antibody prior to secondary staining.

The purified Hu.anti-V5 antibody was shown to be functional in a wide variety of immunological assays, including immunofluorescence (Fig. 8), in which we confirmed that, as predicted, the humanized anti-V5 recognized the W3A strain of PIV5, which has an asparagine (N) at position 100 in P but not a variant with an aspartic acid (D) at this position.

## Discussion

We report here on initial developments of PIV5 lacking the F gene (PIV5ΔF) as a single-cycle vector, a feature that would render the vector safe for the expression of recombinant proteins. We demonstrate the usefulness of this vector in expressing a humanized monoclonal antibody against the V5 epitope. The humanized antibodies with the highest affinity for the V5 tag had a similar high affinity (*K*_D_ in the picomolar range) as the parental mouse mAb. Having shown the successful humanization of the anti-V5 antibody (SV5-Pk1), a similar approach is likely to be successful in grafting the mouse anti-V5 complementarity-determining regions (CDRs) onto the constant domains of other species, thereby generating a suite of antibodies that are likely to have the same binding properties as the original mouse anti-V5 antibody. We envisage that such anti-V5 antibodies may have a number of uses both *in vitro*, when it is inappropriate or problematic to use the mouse anti-V5 antibody (e.g. in immune diagnostics or immunohistochemistry), and *in vivo* (e.g. for the generation of immunogenic SMAA [24] complexes or the targeting of V5-tagged and expressed recombinant marker proteins).

With regard to the use of viral vectors to express recombinant proteins *in vitro*, there are a great number of different expression vectors to choose from, including Sendai virus and PIV2 vectors in which the F gene has been deleted [31,32]. Whilst it is too early to know whether PIV5-based vectors will have a significant advantage over those already developed, they have some important potential advantages. For example, PIV5-based vectors can be engineered to immediately establish persistent infections that do not kill the infected cells [8,9]. In this study, we have only evaluated the use of a PIV5ΔF vector with a phenylalanine at position 157 to produce antibody over a relatively short time period. We did this because although it was predicted to have an acute/lytic phenotype, for reasons that we are currently investigating, the expression of mCherry, as opposed to GFP, somewhat attenuated the acute/lytic phenotype. However, in contrast to PIV5.ΔF vectors with a serine at position 157, i.e. a virus with a ‘true’ persistent phenotype, the expression of mCherry stayed high in all cells infected with PIV5.ΔF.F157.mCherry viruses as they did not flux between active and repressed states over time. However, infection with PIV5.ΔF.F157.mCherry viruses did inhibit the replication of the infected cells, and after 5-day infection, a significant proportion, up to 50%, had died depending on the cell line, in contrast to infection of cells with PIV5.ΔF.S157.mCherry viruses where the vast majority of cells remained alive and could be readily passaged. It will therefore be of interest to compare the advantages and disadvantages of ‘acute/lytic’ vs ‘persistent’ PIV5-based vectors in a variety of situations. For example, if long-term expression is required, both *in vitro* and *in vivo*, persistent vectors may have advantages over ‘acute/lytic’ vectors, but if short-term higher yields of recombinant proteins are wanted, then ‘acute/lytic’ vectors are likely to be better.

Encouragingly, even when low-density cultures of CHO cells were used (compared to optimized conditions where 10^7^–10^8^ cells per millilitre can be achieved), the PIV5ΔF.F157.hu_V5.mCherry expression vector produced ~20–50 mg l^−1^ of the humanized anti-V5 antibody (hu_SV5-Pk1 [6]) after 5 days. Whilst it is not possible to predict the amount of antibody that would be produced under industrial conditions, we note that CHO cell concentrations >1×10^8^ cells per millilitre can be achieved [29]. There are also potential improvements to this technology that might increase both the yield and quality of the antibody produced. For example, the antibody heavy and light chains were encoded from the same synthetic gene, with the TaV T2A sequence inserted between them to ensure that the chains were separated prior to assembly into functional antibody. Whilst this worked well, it has two potential disadvantages. Firstly, an extra 18 aas, derived from the T2A sequence, are attached to the antibody (17 on the C-terminus of the light chain and 1 on the N-terminus of the heavy chain [25,26]). Secondly, we have previously suggested that the longer the mRNA encoded from the PIV5 genome is, the less abundant it will be. This is because there is an increased chance that the virus-encoded vRdRp will fall off the template before the mRNA can be stabilized by the addition of a poly A tail to its 3′ end [33]. We are therefore currently investigating whether the heavy and light chain genes can be driven by separate gene start/stop signals, both negating the requirement for the 2A sequence and potentially increasing the abundance of the heavy and light chain transcripts and thus the yield of antibody. This will also be important if antibodies produced using the PIV5-based vector systems are used for therapeutic purposes as additional residues could be immunogenic and therefore unfavourable. Other potential advantages of using PIV5-based vectors for making antibodies over conventional methods are potentially manyfold: there is no requirement to optimize plasmid ratios as is the case in transfection-based procedures (where heavy and light chain genes are encoded on separate plasmids), it is straightforward to ensure all cells are infected and therefore producing antibody, there is no requirement for cell cloning in order to optimize the levels of heavy and light chain made, as may be required if the genes were inserted by recombinant DNA technology into the host cell chromosome, and PIV5-based vectors can infect virtually any cell type (hence, no specialist expertise or equipment is required for expression). Furthermore, once the recombinant virus has been made, there is very little expense associated with making new batches of antibody, as is the case with transfection methods used for producing antibody or other recombinant proteins.

## Abbreviations

BEFV Gt: bovine ephemeral fever virus glycoprotein
CHO: Chinese hamster ovary
DMEM: Dulbecco’s modified Eagle medium
Dox: doxycycline
F: fusion
HN: haemagglutinin-neuraminidase
Hu: humanized
L: large
Le: leader
M: matrix
NP: nucleoprotein
P: phosphoprotein
p.i.: post-infection
PIV5: Parainfluenza virus type 5
RSV: respiratory syncytial virus
SARS-CoV-2: severe acute respiratory syndrome coronavirus 2
SH: small hydrophobic
SMAA: solid matrix-antibody-antigen
SPR: surface plasmon resonance
St.A.: *Staphylococcus aureus*
SV5: simian virus 5
TaV: *Thosea asigna virus*
Tr: trailer
vRdRp: viral RNA-dependent RNA polymerase
vRNP: viral ribonucleocapsid
